# Kyste arachnoïdien: une cause de lombalgies chroniques à ne pas méconnaitre

**DOI:** 10.11604/pamj.2015.22.235.7251

**Published:** 2015-11-12

**Authors:** Mouna Sghir, Wafa Said

**Affiliations:** 1 Unité de Médecine Physique, CHU Taher Sfar, Mahdia, Tunisia

**Keywords:** Lombalgies chroniques, kyste arachnoïdien, IRM, chronic low back pain, arachnoid cyst, MRI

## Image en medicine

Le kyste arachnoïdien est un diverticule méningé communiquant dans la majorité des cas avec les espaces sous-arachnoïdiens. Il peut avoir une origine congénitale ou traumatique. La présentation clinique dépend de la taille et de la localisation de la formation kystique. Nous rapportons le cas d'une patiente SH âgée de 36 ans, sans antécédents pathologiques particuliers, elle a consulté pour des lombalgies chroniques mécaniques non impulsives évoluant depuis 10 ans et s'exacerbant surtout en position debout. L'examen a trouvé un trouble statique du rachis : une hypercyphose dorsale compensée par une hyper lordose lombaire avec des flèches vertébrales au niveau de C7:100mm, D8: 0mm, L3: 80mm, S: 0mm. Il n'existe pas de syndrome rachidien, le reste de l'examen clinique est sans particularités. Le bilan biologique est revenu normal, la radiographie standard n'a montré que le trouble statique dans le plan sagittal. Devant la non amélioration par un traitement médical et un protocole de rééducation adapté un scanner lombaire a été demandé. L'IRM a conclu à la présence à l'étage L3-L4 d'une masse intracanalaire qui élargit le foramen intervertébral sans signe de compression du fourreau dural en rapport avec un kyste arachnoïdien. Ainsi, la patiente a été adressée en neurochirurgie.

**Figure 1:**
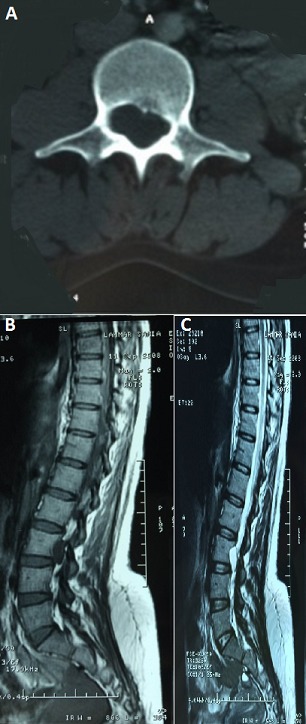
(A) TDM lombaire (coupe axiale): une masse hypo dense élargissant le foramen et exerçant un scalopping sur le mur postérieur de L3; (B) IRM médullaire (coupe sagittale en T1) intracanalaire à l’étage L3-L4 qui élargit le foramen intervertébral sans signe de compression du fourreau dural en rapport avec un kyste arachnoïdien; (C) IRM médullaire (coupe sagittale en T2) intracanalaire à l’étage L3-L4 qui élargit le foramen intervertébral sans signe de compression du fourreau dural en rapport avec un kyste arachnoïdien

